# The potential therapeutic value and application prospect of engineered exosomes in human diseases

**DOI:** 10.3389/fcell.2022.1051380

**Published:** 2022-12-01

**Authors:** Gege Liu, Junlu Wu, Guofei Chen, Anquan Shang

**Affiliations:** ^1^ Department of Laboratory Medicine, Shanghai Tongji Hospital, School of Medicine, Tongji University, Shanghai, China; ^2^ School of Clinical Medcine, Ningxia Medical University, Yinchuan, Ningxia, China; ^3^ Department of Laboratory Medicine, Suzhou Hospital of Integrated Traditional Chinese and Western Medicine, Suzhou, China; ^4^ Department of Laboratory Medicine, The Second People’s Hospital of Lianyungang & Department of Laboratory Medicine, The Oncology Hospitals of Lianyungang, Lianyungang, China

**Keywords:** engineered exosomes, diagnostic, treatment, clinical value, application prospects

## Abstract

Exosomes are tiny vesicles produced by a wide range of cells that contain complex RNA and protein. In the diagnosis, treatment, and prevention of illness, they offer great potential. *In vitro* engineering technique modifies exosomes to produce designed exosomes that include nucleic acids, proteins, and medicines, and are targeted to particular tissues or cells. Their applications range from tumor imaging and gene therapy to vaccine production and regenerative medicine to targeted medication delivery. Many disciplines have promising futures for using this technology. In this review, we’ll look at the potential therapeutic usefulness and use of engineered exosomes in a variety of human illnesses with various systemic manifestations.

## Introduction

Exosomes (Exos) are extracellular vesicles with a diameter of 30–100 nm that are released by a wide range of cells and contain a wide range of biological components such as nucleic acids, lipids, metabolites, and proteins (Pegtel and Gould, 2019). Exosomes originate from the endosomal pathway by the formation of the early sorting endosomes, late sorting endosomes, and ultimately multivesicular body (MVBs), which contain intraluminal vesicles. When MVBs fuse with the plasma membrane, exosomes are released (size range ∼40–160 nm) (Kalluri and LeBleu, 2020). Exos may be found in a wide range of human bodily fluids, including blood, urine, saliva, etc., and other secretions, as well as in immune and cancer cells (Adriano et al., 2021). Other biological functions of Exos include signal transduction, chemical transfer, and angiogenesis promotion. Although Exos come from a variety of sources and contain a wide range of different components, this heterogeneity means that Exos can play a variety of roles in the development of disease and in the diagnosis and treatment of disease (Kalluri and LeBleu, 2020).

Exos are extremely permeable to tissues, blood, and the blood-brain barrier (BBB) (Zheng et al., 2019). Exos from patients have a better histocompatibility and lower toxicity than other medication carriers (Quah and O' Neill, 2005). Exos produced from cells have undergone modifications to enhance their functionality and expand the range of possible therapeutic uses (Yeo et al., 2013). A popular approach is to employ genetic engineering technology to make recipient cells strongly express specific proteins or polypeptides, resulting in designed Exos that are then sent to the membrane surface or the cell cavity of the receiver, **we define modified Exos as Engineered exosomes (Eexos)** (Xu et al., 2021). Based on the molecular transport capacity and targeting characteristics of exosomes, researchers endow exosomes with cell and tissue targeting specificity by modifying exosomes surface molecules, so as to deliver exosomes loaded with protein/RNA/small molecule transporters to the corresponding disease regions or cells (Xu et al., 2021). Target and accumulate in particular cells or tissues by acquiring this substance’s targeting and accumulation capabilities (Xu et al., 2021).

Exosomes are multiple vesicles secreted by most cells, and these exosomes secreted from different cells have the same common components, and studies have shown that there is no mechanism to show that these common components change with the physiological state of cells (Sterzenbach et al., 2017). These exosomes for engineering exosomes can be extracted from a variety of sources, including immune and cancer cell lines, blood, tears, urine, saliva, milk, ascites, eukaryotes, prokaryotes, herbs, etc (Xu et al., 2021).

Exos that have been modified may be loaded with miRNA, siRNA, DNA, medicinal medicines, and other substances and directed to particular tissues or cells to exercise their effects (Ortega et al., 2020). Studies have shown that loading substances into exosomes can be done in a variety of ways, such as fusion, electroporation, ultrasound, co-incubation, and so on. For example, the selected drug is suspended with the exosome under the condition that the drug enters the exosome through the hole generated by the electric field, and an electric field is applied (Gilligan and Dwyer, 2017). The exosome concentration range of each electroperforation is 0.07 - 0.5 μg/μL, which has been shown to be successful uptake at different voltages (Gilligan and Dwyer, 2017). Secondly, engineered exosomes can also be prepared by co-incubating the drug with the exosome if the drug size can pass through the exosome membrane (Gilligan and Dwyer, 2017). Engineered exosomes can also be modified using chemical methods, such as lipidization (Gilligan and Dwyer, 2017). Modified engineered exosomes can be injected intravenously, nasally or intraperitoneally (Nojehdehi et al., 2018; Ortega et al., 2020; Xu et al., 2021).

In addition to the ease with which Exos’ membrane structure may be labeled, a number of engineering methods have been utilized to alter Exos to improve their targeting capabilities, allowing modified Exos to be more useful for illness detection and therapy (Xu et al., 2021). Modified engineered exosomes have been widely used in gene therapy (Bai et al., 2020), targeted drug delivery (Kim et al., 2021), vaccine preparation (Ferrantelli et al., 2018; Di Bonito et al., 2019; Du et al., 2021), regenerative medicine (Liu et al., 2015; Li et al., 2018; Yang et al., 2021), tumor imaging (Srivastava et al., 2018; Chen et al., 2021) and other fields, mainly playing a role of targeted delivery of contents. In this review, we will discuss the clinical use of modified Exos in illnesses affecting many systems in the human body and their potential application to develop novel therapeutic methods for disease detection and treatment.

## Respiratory system

It is possible to use eexos to deliver a wide range of therapeutics, including as miRNA, siRNA, medicines, and more, to patients with a variety of respiratory disorders (Table 1). A great number of experimental research are required to demonstrate Eexos availability and value since most studies have demonstrated that Eexos is largely employed in clinical therapy, particularly cancer. Many discoveries are still in the laboratory stage.

**TABLE 1:** Engineered exosomes in the respiratory system.

Disease type	Encapsulated contents	Function
Lung cancer	AA-PEG-PTXs	Improve anticancer drug efficacy
	miR-449a	Promotes cancer cell proliferation and inhibits cell apoptosis
	siRNA	Interfering RNAs
Lung metastases from breast cancer	miRNA-126	Interrupting the PTEN/PI3K/AKT pathway, inhibiting cancer cell proliferation and metastasis
Acute lung injury/Acute respiratory distress syndrome	hucMSCs-miR-377-3p	Induction of cellular autophagy to improve lung injury
Lung disease	Curcumin-RBP	Reduced proinflammatory cytokines

PTX-Exo can be used to treat mice lung metastases and the addition of aminoethyl anisamide-polyethylene glycol (AA-PEG) to PTX-Exo produced AA-PEG-PTX-exo, which further improved lung cancer therapy (Kim et al., 2018; Bai et al., 2020; Zhou et al., 2021). Non-small cell lung cancer mice were treated with Eexos-miR-449a, a potent exosome with high targeting and delivery efficiency, which suppressed cancer cell proliferation and induced apoptosis to slow tumor growth in the animals (Zhou et al., 2021). It has been shown that a new tLyp-1 exosome may carry siRNA to cancer cells such as non small-cell lung cancer (NSCLC), functioning as a gene silencing therapy (Bai et al., 2020).

Lung metastases are a promising application for Eexos in addition to its involvement in original lung malignancies. Integrin four on the exosome surface recognizes and internalizes breast cancer (MDA-MB-223) Exos, and lung metastasis occurs. However, Exos loaded with miRNA-126 block the cancer cell metastasis-related pathway PTEN/PI3K/AKT and inhibit cancer cell proliferation and metastasis, effectively inhibiting the formation of lung metastasis in a mouse model (Nie et al., 2020; Wei et al., 2020; Kim et al., 2021).

There are two major types of acute lung injury and respiratory failure: acute respiratory distress syndrome (ARDS) and acute lung injury (ALI). One study discovered a novel way to treat lung illness by releasing miR-377-3p from human umbilical cord blood-derived mesenchymal stem cells (hucMSCs) and inducing cell autophagy, which reduced lipopolysaccharide-induced acute lung damage (Wei et al., 2020). To deliver the anti-inflammatory peptide-RBP to the Exos, it bound to exosomal membrane integrins and formed an RBP-Exos complex with anti-inflammatory properties. Curcumin loading into RBP-Exo (Cur-RBP-Exos) enhances curcumin transport efficiency and enhances curcumin’s anti-inflammatory activity. When given to mice with acute lung damage, RBP-Exo supplemented with curcumin decreased the production of pro-inflammatory cytokines and controlled the inflammatory response more efficiently (Kim et al., 2021).

Lung cancer is the cancer with the highest mortality rate in the world. Eexos have been used in the treatment of lung cancer by loading drugs, miRNA, siRNA, etc., to enhance the therapeutic effect. In addition, engineered exosomes have been investigated for the treatment of other respiratory diseases. Modified engineered exosomes play a therapeutic role by inhibiting the growth and proliferation of tumor cells, regulating tumor-related signaling pathways, regulating immune cells, and targeting drug delivery.

## Digestive system

Because of the digestive system’s complexity and the wide range of disorders it might affect, Eexos has continuously improved its therapy capabilities for those conditions (Table 2). Diagnosis and therapy of digestive system malignancies in complicated systems benefit greatly from using Eexos’ highly focused ability to precisely detect the lesion location and prevent harm to normal tissues.

**TABLE 2:** Engineered exosomes in the digestive system.

Disease type	Encapsulated contents	Function
Colorectal cancer	5-FU/miR-21i	Improve cancer cell resistance
	Oxaliplatin/PGM5-AS1	Enhanced efficacy of oxaliplatin and reduced tumor metastasis
Liver cancer	HepG2	Caes specific lysis of HCC cells
	Erastin/Rose Benga	Induced HCC cell death
Pancreatic cancer	siRNA	Targeted mutation sites in cancer cells
	Survivin-T34A	Increased cancer cell apoptosis
Oral squamous cell carcinoma	siLCP	Inhibition of tumors

Anti-cancer medicine 5-fluorouracil (5-FU) and miR-21 inhibitor oligonucleotide (miR-21i) were loaded into Exos (5-FU/miR-21i-Exos) from colorectal cancer cells HCT-116 and then delivered to the cells *via* Exos (Liang et al., 2020a). According to the findings, the Exos effectively promoted cancer cell uptake of the anti-cancer drug 5-FU and miR-21i, and significantly reduced the expression of miR-21, which mediates drug resistance, and enhanced the cytotoxicity of 5-FU for cancer cells. This suggests Eexos offers a therapeutic approach for drug resistance in cancer cells (Liang et al., 2020a). Cancer cells that have acquired oxaliplatin resistance can no longer be treated with oxaliplatin. Studies have shown that Exos encapsulating oxaliplatin and PGM5 antisense RNA (PGM5-AS1) (oxaliplatin/PGM5-AS1-Exos) can reverse cancer cell resistance, increasing the effectiveness of oxaliplatin and effectively inhibiting cancer cell metastasis (Hui et al., 2021). Oxaliplatin resistance is common in metastatic cases of colorectal cancer.

Dendritic cells activated by tumor lysate have been shown to stimulate immune responses in mice, slow tumor growth, and aid in the treatment of hepatocellular carcinoma. This study found that human HepG2 cell-derived Exos stimulated immune responses in mice, which improved the tumor microenvironment, resulting in specific lysis of hepatitis C tumor cells. Delivery of Erastin Er and Rose Bengal RB, two drugs that cause iron death in tumor cells, to the liver is an efficient way to kill liver cells (Rao et al., 2016). When Erastin and Rose Benga were loaded into Exos using ultrasound, it formed Er/RB-ExoCD47, which had CD47 molecules on the exosome surface. This allowed the Exos to avoid being phagocytosed by the mononuclear phagocytic system. When mice are injected with drug-laden Exos, only hepatocellular carcinoma cells die, while no other organs are harmed (Du et al., 2021).

One of the most important risk factors for pancreatic cancer is a GTPase KRAS mutation. Short interfering Eexos designed to target mutant sites in pancreatic cancer in normal fibroblast-like mesenchymal cells dramatically inhibited pancreatic cancer in mice, enhanced survival and offered a novel strategy to target mutated sites for pancreatic cancer therapy in mice (Aspe et al., 2014; Anderson and Morrow, 2017; Kase et al., 2021). Tetracycline-modified and loaded Exos from human melanoma cells were used to study the effects of an apoptosis inhibitor protein dominant inactivating mutant on pancreatic cancer cell growth in mice. This mutant, Survivin-T34A, was found to significantly increase apoptosis in the pancreatic cancer cell line, suggesting that it could be used to treat pancreatic cancer (Aspe et al., 2014). Through the use of an electroporation approach, Exos that are specifically targeting oral squamous cell carcinoma cells may be loaded with siLCP1 (siLCP1-Exos) and delivered to the cancer cells to inhibit them (Kase et al., 2021).

The incidence and mortality rates of liver cancer, colorectal cancer and gastric cancer in the digestive system are among the first few. Targeted delivery of therapeutic drugs and siRNA by Eexos can regulate the occurrence and development of tumors, immune microenvironment and tumor drug resistance, which provides a new idea for the treatment of digestive system cancers.

## Cardiovascular system

Cardiovascular illnesses continue to be among the world’s deadliest and most debilitating conditions, and Eexos therapy may help protect the heart, lessen the severity of the disease, and lessen the consequences it causes (Table 3).

**TABLE 3:** Engineered exosomes in the cardiovascular system.

Disease type	Encapsulated contents	Function
Atherosclerosis	HAL	Enhanced anti-inflammatory effects of HAL for fluorescence imaging of hardened plaques
Acute myocardial infarction	miR-93-5p	Protection of the infarct-induced myocardial injury
	IMTP	Targeted ischemic myocardium to treat myocardial infarction
	MIF	Enhance cell proliferation, angiogenesis, and inhibit cardiomyocyte apoptosis
Ischemic-Reperfusion Injury	miR-125b	Targeted SIRT7 to mitigate myocardial injury
	Curcumin	Neurovascular vessels were recovered after ischemia and reperfusion

Heart disease is caused by atherosclerosis, which may be effectively treated by decreasing inflammation. A research has shown anti-inflammatory properties in Exos generated from macrophage M2, including the ability to bind anti-inflammatory substances released by M2. It was discovered that electroporating HAL (hexyl 5-aminolevulinic acid hydrochloride) into the M2-derived Exos (HAL-Exos) improved the anti-inflammatory effects and reduced atherosclerosis by using HLA’s potential to create anti-inflammatory carbon monoxide and bilirubin. Another way in which HLA contributes to heme production is *via* protoporphyrin IX (PpI IX), an intermediate metabolite that allows for fluorescence imaging of atherosclerosis (Wu et al., 2020).

It is critical to recognize and treat acute myocardial infarction early in order to prevent mortality and morbidity (Anderson and Morrow, 2017). It is a frequent cardiac emergency. Studies have revealed that ADSCs secreting miR-93-5p-loaded Exos (miR-93-5p-Exos) from adipose-derived stromal cells protect against infarct-induced cardiac damage more effectively (Liu et al., 2018). After a heart attack, the use of Exos fused to the ischemia cardiac targeting protein (IMTP) has been shown to have a therapeutic impact on heart attack victims with an acute myocardial infarction (Wang et al., 2018). It was found that loading the macrophage migration inhibitory factor (MIF) into human umbilical cord blood MSC-derived Exos, known as MIF-Exo, increased proliferation and angiogenesis in the human umbilical vein endothelial cells (HUVEC) and inhibited apoptosis, showing good cardioprotective effects in an acute myocardial infarction rat model (Zhu et al., 2021).

Exos generated from bone marrow mesenchymal cells containing miR-125b (miR125b-Exos) have been shown in a rat model to reduce cardiac tissue damage by focusing on SIRT7 and exerting cardioprotective effects. In the case of ischemia-reperfusion damage, the complex generated by mixing embryonic stem cell Exos with curcumin may aid neurovascular repair by achieving effective therapeutic effects (Kalani et al., 2016).

To sum up, Eexos have been used to treat common cardiovascular diseases such as atherosclerosis, acute myocardial infarction and ischemia-reperfusion injury by regulating inflammatory response and immune cells to target the delivery of drugs (such as curcumin).

## Genitourinary system

The whole urinary system may be affected by diseases of the kidneys, bladder, prostate, and other organs. Eexos containing therapeutic medications are increasingly being used to treat illnesses effectively (Table 4).

**TABLE 4:** Engineered exosomes in the genitourinary System.

Disease type	Encapsulated contents	Function
Kidney injury	miRNA-let7	Upregulated gene expression in the damaged kidney
Acute kidney injury	MSC-Exos	Anti-inflammatory, reducing AKI caused by chemotherapeutic agents
	MSC-Exos-PRP	Activation ating the AKT/Rab27 pathway to repair AKI
Renal Cell Carcinoma	miR-224-5p	Treatment targets for renal cell carcinoma
Prostate cancer	si-RNA	Silencing of SIRT6, inhibits the proliferation of cancer cells and metastasis

MiRNAs may be used to treat a damaged kidney. Gene expression may be regulated selectively by MSCs overexpressing miRNA-let7, homing to the injured kidney to upregulate. This procedure is mostly carried out in mice by the MSCs secreting Exos (miRNA-let7-Exos), which act on the recipient cells. To repair damaged kidneys, researchers are now using modified MSC-secreted Exos that carry miRNAs to specific locations in the body (Wang et al., 2016).

It is typical for hospitalized patients to suffer from acute kidney injury. MSCs are regarded an excellent treatment for tissue damage, and Exos generated from MSCs may be used to treat acute kidney injury. Researchers found that MSC-Exos were more efficiently taken up by tubular epithelial cells in a mouse model of acute kidney injury and showed efficient anti-inflammatory capacity, showing great potential in the treatment of acute kidney injury caused by chemotherapeutic agents using a three-dimensional culture system with a hollow fiber bioreactor (Cao et al., 2020). Serum urea nitrogen and creatinine levels may be dramatically reduced by using PRP-MSCs, as well as the histopathological kidney damage reversed. Exos produced from adipose tissue-derived MSCs rescue mice from acute renal damage (Gao et al., 2020a).

Cancer-associated fibroblasts (CAF) in the cancer stroma release Exos that may impact tumor growth in clear cell renal cell carcinoma. Exos from CAF were shown to transport miR-224-5p (miR-224-5p-Exos) into renal cell carcinoma cells, increasing the course of the disease. This suggests that miR-224-5p might be a therapeutic target for renal cell carcinoma (Liu et al., 2021). In another work, Exos loaded with siRNA silenced SIRT6 *in vitro* and *in vivo*, resulting in SIRT6 deletion and a substantial reduction in prostate cancer cell growth and metastasis in mice (Han et al., 2021).

## Gynecology

Gynecological disorders, particularly cancers of the uterus and fallopian tubes, have grown to be a significant threat to women’s health. Cancer cell proliferation and invasion may be inhibited by using Exos, which regulate hormone secretion in the body, regulate the tumor microenvironment, and regulate hormone secretion *via* the gynecological system (Table 5).

**TABLE 5:** Engineered exosomes in the gynecology.

Disease type	Encapsulated contents	Function
Endometrium carcinoma	HCG-Exos	Targeted therapy
Ovarian cancer	CRISPR-Cas9	Induced apoptosis in cancer cells and enhance the efficacy of chemotherapeutic drugs
	peptide	Overexpressing of the miR-92b-3p and targeting of the vascular endothelial cells
	miR-199a-3p	Targeted c-Met expression of cancer cells inhibited tumor proliferation and invasion
Breast cancer	siRNA	Targeted TD52, gene therapy, and drug delivery
	miR-let-7a	Target EGFR, Inhibition of tumor progression

There are studies showing that Human Chorionic Gonadotropin (HCG)-carrying Exos can be delivered into the endometrium to increase endometrial tolerance by loading HCG into the endoscopic Exos through freeze-thaw cycles and ultrasound treatment, and that endoscopic Exos can also carry endometrial therapeutic agents to achieve targeted endometrial treatment (Hajipour et al., 2021).

To trigger apoptosis in ovarian cancer cells and increase the susceptibility of ovarian cancer cells to cisplatin-based chemotherapeutic drugs, cancer cell-derived Exos may convey the CRISPR-Cas9 system to SKOV3 cells (Kim et al., 2017). Exos derived from ovarian cancer cells increased the angiogenic and migratory capacity of vascular endothelial cells in an unrelated mouse model, which promoted tumor-associated angiogenesis. This showed the potential of artificially generated Exos overexpressing miR-92b-3p as anti-angiogenic agents, which may provide a new approach for the treatment of ovarian cancer that is anti-angiogenic. Using Exos and peptides to create miR-92b-3p overexpressed peptide-engineered Exos (miR-92b-3p-Exos) that target vascular endothelial cells to decrease angiogenesis and tumor development To test whether Eexos may be utilized for targeted treatment, researchers employed Exos loaded with miR-199a-3p to decrease tumor growth and invasion in a mouse model of ovarian cancer. (Kobayashi et al., 2020; Wang et al., 2021).

Exos loaded with siRNAs targeting TD52 are delivered to HER2-positive breast cancer cells to modulate the expression of the TD52 gene for gene therapy and medication delivery (Limoni et al., 2019). Breast cancer is now the most common cancer in women. Exos may carry miRNAs in the form of miR-Exos, which are loaded into cells. Intravenous administration of miR-let-7a-loaded Exos to mice had inhibitory effects on tumor development in the presence of breast cancer cells expressing the epidermal growth factor receptor (EGFR) (Ohno et al., 2013).

Women ovarian cancer, breast cancer, the male urogenital system such as prostate cancer, kidney cancer incidence continues to increase, research shows that Eexos shows great potential in cancer treatment, development by regulating tumor cells, and the ingredients in the tumor microenvironment, compared to the pure drug therapy, further enhance the therapeutic effect.

## Nervous system

The central nervous system (CNS), which includes the brain and spinal cord, is the body’s top organ for regulating and controlling numerous functional processes. The peripheral nervous system connects it to other bodily systems and organs. Because many medications cannot cross the blood-brain barrier to treat CNS diseases, therapy options are restricted (Srikanth and Kessler, 2012). Many biological barriers may be crossed by Exos, making drug-loaded Eexos a promising therapy option for CNS diseases, according to research (Srikanth and Kessler, 2012) (Table 6).

**TABLE 6:** Engineered exosomes in the nervous system.

Disease type	Encapsulated contents	Function
Brain disease	Gold nanocages	Promote neuronal cell access to exosomes
	AAV	Reduce the antibody concentration after the application of the AAV vector
Parkinsons disease	Catalase-miRNA	Reducof neurotoxicity and neuroinflammation
Epilepsy	MSC-exos	reducing hippocampal neuroinflammation, and reduce neurosis and abnormalities
Glioma	SPIONs/curcumin	Combined with target proteins to facilitate the diagnosis and treatment of glioma imaging
	miRNA-29a-3p	Reduce glioma metastasis and reduce angiogenesis
Brain metastases	CXCR4/TRAIL	Treatment of brain metastases due to breast cancer
Acute ischemic brain injury	MESC-Eur	Treatment of neurovascular injury
The central nervous system	IFN	Anti-inflammatory and neuroprotection
Nerve Injuries	NT-3	Promote nerve regeneration and improve muscle repair

Medications may have a hard time getting into the brain because of the blood-brain barrier. Anticancer medicines delivered through brain endothelium-derived Exos decreased cancer cell fluorescence and tumor growth indicators considerably, demonstrating that Exos may transfer pharmaceuticals across the blood-brain barrier and display beneficial therapeutic effects as a drug delivery mechanism (Yang et al., 2015). The blood-brain barrier may be repaired using endothelial colony-forming cells (ECFC) obtained from human umbilical cord blood. There is evidence to suggest that tissue-resident endothelial cells (EC) produced from the ECFC increased proliferation and migration of this kind of EC in mice’s brain, therefore repairing the damaged blood-brain barrier. Certain origin Exos have been shown to pass the blood-brain barrier and have favorable impacts on the blood-brain barrier’s overall integrity (Gao et al., 2018).

Using gold nanoparticles, researchers loaded markers into bone marrow mesenchymal cell-derived Exos (MSC-exos) and tracked the distribution of these Eexos in mice with various neurological disorders such as Parkinson’s, Alzheimer’s, and stroke using conventional X-ray computed tomography CT techniques. The results showed that MSC-Exos can be acquired by neuronal cells in disease areas to track the severity of the condition and can be used for targeted drug (Perets et al., 2019). Toxicities of adeno-associated virus (AAV) can be reduced by combining Exos with AAV, and this combination can also slow the rapid clearance of AAV vectors. Moreover, expression of brain-targeting peptides on the surface of Exos can improve transduction, and this can help reduce the amount of anti-AAV antibodies produced following AAV administration (György et al., 2014).

Neuroinflammation has been linked to Parkinson’s disease, which is a degenerative condition. To minimize neurotoxicity and inflammation, Exos loaded with catalase mRNA may be delivered to the brain (Kojima et al., 2018). Using MSC-exo has been shown to have anti-inflammatory effects, and after 6 h of intranasal injection of MSC-exo into epileptic mice, it was found that Exos reached the hippocampus, where they reduced inflammation and prevented neurodegeneration and abnormalities from arising in the hippocampal region (Long et al., 2017).

Treatment for glioma is complicated because the blood-brain barrier prevents therapeutic drugs from entering the brain. Loading SPIONs and curcumin into Exos (SPIONS/Cur-Exos), which can easily cross the blood-brain barrier and bind Exos to target proteins chemically, offers a new approach to the diagnosis and treatment of glioma (Jia et al., 2018). Exos overexpressing mesenchymal stem cell miRNA-29a-3p can diminish glioma metastasis and angiogenesis by inhibiting their migration and angiogenesis *in vitro* (Zhang et al., 2021).

A new therapeutic strategy for treating brain metastases caused by breast cancer may be formed by using genetic engineering to prepare CXCR4/TRAIL Exos, which can selectively induce apoptosis in cancer-transformed cells. These Exos can cross the blood-brain barrier (BBB) and can be used to treat brain metastases (Liu et al., 2020). Acute ischemic brain damage activates vascular endothelial cells, which may then stimulate neural progenitor cell proliferation and migration. Vascular endothelial cells (VEC) Exos stimulate proliferation and migration of neural progenitor cells and decrease apoptosis, indicating that Exos are critical for neuronal reconstruction and brain protection in acute ischemia damage (Gao et al., 2020b; Zhou et al., 2020). When embryonic stem cells loaded with curcumin were used to treat cerebral ischemia-induced neurovascular loss, it was shown that this MESC-exo helped in the regeneration of the brain’s blood vessels (Kalani et al., 2016). Researchers put Neurotrophin-3(NT-3) miRNA into adipose stem cell ADCS-derived Exos, which were then loaded into nerve-guided catheters in rats for the repair of neurological abnormalities. NT-3 is a chemical that contributes to peripheral nerve regeneration. AdCS-exo enhanced nerve regeneration and improved muscular recovery in rats, according to the findings (Yang et al., 2021).

## Bone and joint system

Various complicated motor functions in the human body include the bone and joint system, which has a crucial role to play in preserving the body’s structure and function. Bone and joint illnesses, as well as bone and joint restoration, may benefit from the use of exos, which contain medications or gene expression modulators (Table 7).

**TABLE 7:** Engineered exosomes in the bone and joint system.

Disease type	Encapsulated contents	Function
Osteoarthritis	miR140	Treatment of cartilage defects
	KGN-E7	Targeted MSC to treat cartilage degeneration
Segmental bone defects	VEGF	Induced MSC osteogenic differentiation of MSCs and remodeling of blood vessels
Tendon injury	miR-29a-3p	Promote tendon regeneration
Degenerative disorder	peptide	Enhance the stability and efficacy of drugs, and accelerate muscle growth

MiRNAs that target chondrocytes in Exos may be utilized to treat cartilage damage using eexos produced from these miRNAs. Exos expressing the cartilage-affinity peptide CAP on their surface may be used to convey miR-140-loaded Exos to deep cartilage areas, according to research. Interarticular injection of CAPExoMiR140 can be used to treat cartilage abnormalities than unmodified Exos, thereby slowing osteoarthritis development, and might be employed as an innovative cell-free therapy (Liang et al., 2020b). Studies have shown that MiR-214 regulates osteoblast function by targeting specific molecular pathways and the expression of various osteoblast-related genes, while promoting osteoclast activity by targeting phosphatase and tensin homolog (Pten) (Sun et al., 2018). The exosomal miRNA paracrine mechanism is used to mediate osteoclast-osteoblast intercellular crosstalk, and osteoclast-derived exosomal miR-214 can be transferred to osteoblasts to inhibit bone formation, which can be used to treat bone diseases with reduced bone formation (Li et al., 2016; Zhu et al., 2018).

Osteoarthritis cartilage degradation may be treated by mesenchymal stem cells from synovial fluid (SF-MSC). To induce SF-MSC differentiation into chondrocytes, researchers used Kartogenin (KGN). KGN bound to E7-Exo, a peptide that targets MSC, forming Exos that target MSC and delivering it to the surface of SF-MSC to promote differentiation. This produced more significant therapeutic effects and made KGN an advanced stem cell therapy (Kim et al., 2017). When ATDC5-derived Exos are used to make Eexos, they may be used to stimulate osteogenesis and vascular regeneration in MSCs. These Exos can release VEGF genes, which can then be used to remodel the vascular system and aid in vascularized bone repair (Zha et al., 2021). Treatment of tendon injuries using exosomal HUMSC-Exo from human umbilical cord blood MSCs has been shown to be more successful, and genetically editing HUMSC-Exo overexpressing miR-29a-3p has also suggested that Eexos may be employed (Yao et al., 2021). Functional dystrophin deficiency causes muscle atrophy in people with Duchenne muscular dystrophy (DMD). The serum stability and efficiency of the pre-muscle growth inhibitor peptide are much improved by attaching it to the surface of Exos. Exos attached with myostatin pro-peptide given to mice on a regular basis promote muscle development, offering a novel medication delivery mechanism for the treatment of DMD (Ran et al., 2020).

Eexos also have great application potential in regenerative medicine, such as inducing osteogenic differentiation and vascular remodeling of bone marrow mesenchymal stem cells, promoting tendon regeneration and so on, which broaden new ideas for the treatment of bone and joint diseases.

## Immune system

Multiple body systems are affected when the immune system is weakened because of the immune system’s role in immunological surveillance, defense, and regulatory actions. It's possible that eexos-mediated immune system regeneration might be a novel way to boost treatment’s effectiveness (Table 8).

**TABLE 8:** Engineered exosomes in the immune system.

Disease type	Encapsulated contents	Function
Rheumatoid arthritis	DS	Promote M1-M2 polarization and reduce inflammation
	ROS-reactive tolD	Downregulation of cytokine expression, and anti-inflammatory
SLE	curcumin	Inhibition of macrophages in producing inflammatory factors
Body lymphedema	VEGF-CD63	Promote lymphovascular endothelial cell proliferation and migration
HIV	ZEP-362	Inhibition of HIV1 expression

In the case of rheumatoid arthritis (RA), MSC-Exos was investigated as a potential anti-rheumatic drug. When Exos derived from DS-modified MSCs are administered to inflamed areas of RA, they reduce inflammation and block the activity of pro-inflammatory cells, leading in a novel cell-free therapeutic method for the treatment of RA. RA may be lessened by intra-articular autotolerant dendritic cells (tolDC) (Bell et al., 2017). It is possible that ROS, as a physiological stimulus in the RA microenvironment, can alleviate the severity of the disease and represent a new therapeutic approach by inducing the T-cell differentiation center (tlDC) to release Exos that reduce pro-inflammatory factor secretion (Lee et al., 2021). Human immunodeficiency virus (HIV) Research shows that HIV causes immune system abnormalities and a zinc-lipid protein (ZEP-362) targets the HIV-1 promoter, reduces HIV epigenetic expression and limits viral infection by constructing Exos that encode a zinc-lipid protein for HIV-1 and encapsulate it to decrease viral expression (Kalani et al., 2016).

## Endocrine system

Multiple substances interact to exert regulatory effects on the endocrine system’s functions (Table 9). There are substantial health consequences to untreated diabetes mellitus, which is an endocrine system disorder characterized by inadequate insulin secretion or activity.

**TABLE 9:** Engineered exosomes in the endocrine system.

Disease type	Encapsulated contents	Function
Diabetic Wound	LncRNA-H19	Neutralization of inhibition of vascular regeneration due to hyperglycemia
	miR-31-5p	Promote angiogenesis, reepithelialization
	miRNA-221-3p	Promote skin wound healing
autoimmune type 1 diabetes	AD-MSC-Exos	Increasing the number of regulatory T cells plays an immunomodulatory role
Diabetic Peripheral Neuropathy	miR-146a	Inhibiting the TLR-4/NF-κB signaling pathway and inhibiting peripheral blood monocyte activation

In diabetic individuals, LncRNA-H19 levels were shown to be considerably lower, and this may have an effect on angiogenesis. Diabetic wounds are a typical consequence of diabetes. Therapy delivery of LncRNA-H19-exo dramatically accelerated wound healing, indicating that LncRNA-H19-exo has the potential to counteract hyperglycemia and limit vascular regeneration, giving an efficient therapeutic impact on diabetic wounds (Tao et al., 2018). These mesenchymal stem cells have immunomodulatory effects on T-cell inflammation and may enhance autoimmune responses when administered as Exos. T1DM in a mouse model has been demonstrated to be improved by boosting regulatory T cells and their products without modifying the lymphocyte proliferation index, which makes AD-MSC-exo a more effective and realistic technique. AD-MSC-exo has been proven to improve autoimmune T1DM (Nojehdehi et al., 2018). A common complication of diabetes is diabetic peripheral neuropathy, and studies using Eexos loaded with miR-146a and injected into mice found that miR-146a-exo significantly inhibited the activation of peripheral blood nucleated cells by inhibiting the TLR4/NF-B signaling pathway, helping to relieve peripheral neurovascular dysfunction and improve functional recovery (Fan et al., 2021).

## Hematological system

Eexos may be used to deliver therapeutic medications that block the development of a class of illnesses characterized by anomalies in the blood and hematopoietic organs, therefore postponing disease progression and introducing a novel therapeutic method into clinical practice (Table 10).

**TABLE 10:** Engineered exosomes in the hematological system.

Disease type	Encapsulated contents	Function
CML	Imatinib/BCR-ABLsiRNA	Targeted onoblasts to inhibit tumor cell growth
B-CLL	GP350	Teting malignant B blasts to stimulate specific T cells to an immune response

Chronic myeloid leukemia (CML) parent cells overexpress IL-3 receptors in chronic myeloid leukemia CML. We fused IL-3 with Exos containing chemotherapeutic agents or BCR-ABL-siRNA and targeted the fusion Exos to CML mother cells in order to more effectively limit cancer cell proliferation and give improved therapeutic effects (Bellavia et al., 2017). By encapsulating GP350 in Exos, this Eexos targets malignant B-blast cells in patients with B chronic lymphocytic leukemia, conferring on malignant B-blast cells the immunogenicity of EBV, stimulating EBV-specific T cells to generate an immune response, and thereby providing a novel immunotherapeutic approach for B- Chronic lymphocytic leukemia (B-CLL) (Ruiss et al., 2011).

Compared with other systemic diseases, diseases of the immune system, endocrine system and blood system require less traumatic treatment methods such as drug regulation, molecular therapy and cell therapy. Exos can be loaded with corresponding drugs and molecules to enhance their targeted delivery, so as to enhance therapeutic effect and reduce side effects.

## Others

HPV16-E7 is a human papillomavirus (HPV) tumor-associated antigen that activates cytotoxic T-lymphocytes (CTLs). Endogenous Eexos is created by coupling Exos with HPV16-E7 and administering it intramuscularly to produce an efficient anti-HPV16-E7 immune response. This suggests that Eexos may be exploited for vaccine development (Di Bonito et al., 2019) (Table 11).

**TABLE 11:** Engineered exosomes in the other diseases.

Disease type	Encapsulated contents	Function
peritoneal metastatic nodules	gETL NPs	Exosomes improve the disease by the application of intraperitoneal hyperthermia and chemotherapy
Gingival diseases	IL-1RA	Maintain the rapid healing of the wound
HPV vaccine	HPV-E7	Activation of a potent anti-HPV16-E7 immune response

## Conclusion

**FIGURE 1:**
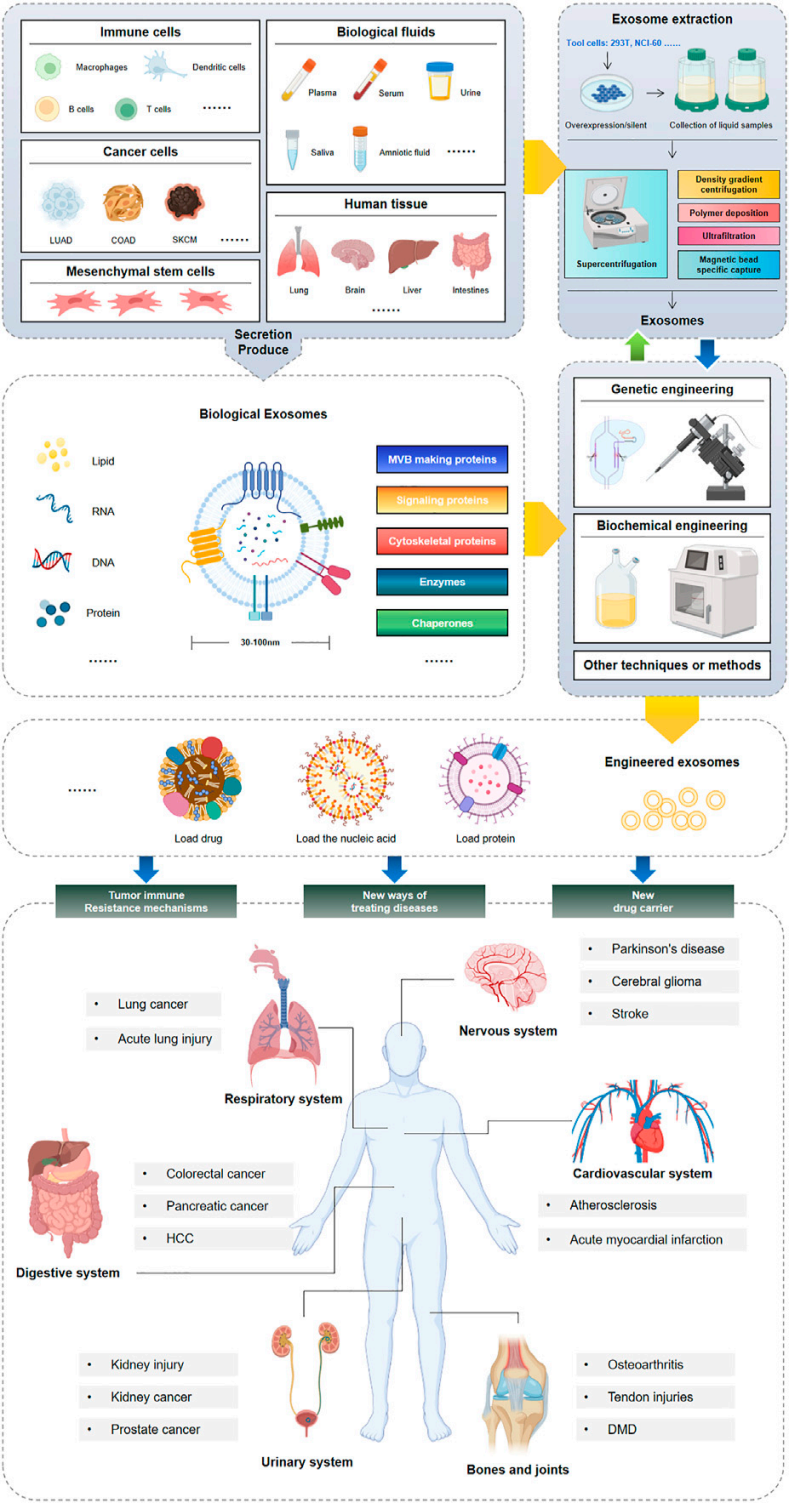
Engineering exosomes preparation and application.

There are several methods to employ Exos in the human body, including illness detection, vector transfer, disease therapy, and liquid biopsy. Exos are found in a wide range of cells and bodily fluids and have become a major source of disease biomarkers. Biochemical engineering, physical engineering and other engineering technologies can process and modify Exos to load miRNA, siRNA, proteins and other substances for tumor imaging, gene therapy, targeted drug delivery and vaccine preparation as well as non-cytotoxicity, low immunogenicity and other fields (Figure 1). It's becoming a popular method for both diagnosing and treating illness. Eexos, a cell-free therapy for cancer, has shown remarkable promise and usefulness in various studies, especially in the area of cancer. The human body is a complex system, and Exos play a part in many of these systems, highlighting the relevance of Exos and the necessity to continue our research in specific areas like the genesis and function of Exos in order to better understand this important cellular component.

Despite the fact that Eexos has a lot of promise in a wide range of disciplines, there are still numerous obstacles to overcome. Since stable Exos are critical to the production of Eexos, the extraction and purification of Exos remain a difficult problem to solve for the time being. Another problem is that Eexos is still in the experimental stage, which means further clinical trials are required to demonstrate its efficacy in human applications. To sum it up, if Eexos is to be utilized in the clinic, it will need to be studied further in terms of cost and affordability, as well as how the Exos are supplied, their half-life *in vivo*, and their negative effects when combined with other medications.

However challenging it may be, Eexos has a lot of potential in the field of illness therapy. With the benefits that Exos have over other nanocarriers, further research into Eexos for clinical applications is needed to improve their clinical relevance and usefulness in the future. Exosome loading efficiency, such as lowering drug loss, enhancing usage, and reducing contamination during reproduction, may still be improved. However, in clinical applications, undesirable effects or side effects of Exos must also be taken into account. All things considered, Eexos has tremendous promise for use in the diagnosis and treatment of illness, as well as in future clinical trials.
